# The New Era of Womanhood

**Published:** 1885-10

**Authors:** 


### The New Era of Womanhood.

The "renaissance of womanhood," as Mrs. Sutherland Orr calls it, is demonstrated by the large number of the gentler sex who pour into our cities and manufacturing towns by the early trains every day. It is regarded as a sign of progress, and comparisons are drawn with the time when, to quote from a Boston daily : "The women who are now crowding and over-crowding the labor market stayed quietly at home, economized rather than earned, endured poverty, neglect, privation in every form of material and spiritual interest, and, for the most part, endured it in silence and seclusion. If a woman did not marry she was probably a more or less unwelcome hanger on, either as 'the last ungathered rose' of her ancestral tree, or in the home of family relatives, where she accepted uncomplainingly burdens of service far greater than those that in this city yield her a comfortable and independent income. Then men went abroad to find their career; women stayed at home awaiting their only career, marriage to find them. Now, in every city and town, women are crowding to the business centers."

It may be well, however, to carefully consider this exodus from the farms to the cities, I am firmly convinced that the reason why more young men now do not determine to be farmers, as their grandfathers and fathers were before them, is that the girls do not fancy becoming farmers' wives. Those who do marry farmers have it understood that they are not to milk, or make butter and cheese, or take care of the poultry, and they are to have a hired girl to do the household work.

Strong, healthy girls turn with disdain from housework and the dairy on a farm to go to a city, where they wearily stand all day behind counters, or run fatigueing sewing machines until the roses leave their cheeks and they become consumptive, pale, hollow-cheeked, prematurely old women. They can dress more showily than they could have done in the country, I admit, and too often they are led into the broad, down-grade road of dissipation, where degradation is ended with welcome death.

Would it not be better, instead of having this tide of young women sweeping into cities and large towns to keep them on the farms, finding pleasant employment for them there? I will admit that the olden time farmer's wife had to slave herself, but then she was hearty, healthy and happy. Why not make the labor of women-folks in the country lighter, and keep them there? It is a topic worthy of consideration. If there is to be a "renaissance" of women," let them be "born again" in the country, and not amid the temptations and the vices of the city or manufacturing towns.—Ben Perley Poore in *American Cultivator*.

